# Association between MTTP genotype (-493G/T) polymorphism and hepatic steatosis in hepatitis C: a systematic review and meta-analysis

**DOI:** 10.1186/s12944-023-01916-x

**Published:** 2023-09-19

**Authors:** Xiaoxia Wang, Yu Cao, Jia Guo, Dezhao Li, Haitao Zhang, Qingkun Song, Jun Lu

**Affiliations:** 1 Department of Medical Oncology, Beijing YouAn Hospital, Capital Medical University, Beijing, China; 2 Department of Clinical Epidemiology Research, Beijing YouAn Hospital, Capital Medical University, Beijing, China; 3 Department of Hepatobiliary Surgery and Liver Transplantation Center, Beijing YouAn Hospital, Capital Medical University, Beijing, China

**Keywords:** MTTP, Genotype polymorphism, Hepatitis C, Hepatic steatosis

## Abstract

**Background:**

Hepatitis C has been associated with the development of hepatic steatosis, which increases the risk of liver cancer. The microsomal triglyceride transporter protein (MTTP), is a lipid transport protein that mediates lipid metabolism and CD1d antigen presentation. The study aimed to explore the association between MTTP genotype (-493G/T) polymorphism and hepatic steatosis in hepatitis C.

**Methods:**

The database “Pubmed, Cochrane library, CNKI, Web of science, Embase and CBM” were retrieved to identify the literature. The quality of the selected literature was evaluated using the “the Newcastle–Ottawa Scale” (NOS). Relevant data was extracted and analyzed using the Stata software. Heterogeneity was expressed by “Cochran's Q and I^2^”, with I^2^ ≥ 50% or *P* < 0.05 indicating high heterogeneity. A random-effects model and subgroup analysis were conducted to identify the sources of heterogeneity. We also used “Funnel plots”, “Egger’s tests” and “Begg’s tests” to evaluate biases in the literature.

**Results:**

The study found a significant and positive association between liver steatosis and the HCV genotype 3 with a dominant model of the MTTP genotype (-493G/T) (OR = 11.57, 95%CI: 4.467–29.962, *P* < 0.001). In contrast, no correlation was found between hepatic steatosis and either the recessive, homozygous or heterozygous models (OR = 1.142, *P* = 0.5; OR = 1.581, *P* = 0.081; OR = 1.029, *P* = 0.86). There was no significant publication biases, as measured by the Funnel plot, and the Egger’s and Begg's tests. Finally, sensitivity analysis showed the obtained results are stable.

**Conclusions:**

Dominant mutations in the T allele of the MTTP genotype (-493G/T) increase susceptibility to hepatic steatosis in patients presenting with the HCV genotype 3.

**Supplementary Information:**

The online version contains supplementary material available at 10.1186/s12944-023-01916-x.

## Introduction

Hepatitis C virus (HCV) infection causes persistent hepatic inflammation that becomes chronic in ≥ 80% of infected individuals, of which 20–30% experience further progression to cirrhosis and liver cancer [1]. Chronic hepatitis C is a systemic disease involving disturbed lipid metabolism and oxidative stress. An evaluation using liver biopsy usually shows hepatic steatosis and fibrosis as the most frequent histological changes [2]. Previous studies suggested HCV patients more easily to develop hepatic steatosis than those presenting with other liver diseases [3, 4]. In turn, persistent hepatic steatosis increases the risk of hepatocellular carcinoma (HCC) [5, 6], which highlights the need for early detection and clinical intervention.

The available literature on the factors contributing to susceptibility to hepatic steatosis associated with hepatitis C shows both the viral genotype and the genetic susceptibility of the host are relevant for this outcome [7–9]. Previous studies found patients carrying the genotype 3 of HCV are more likely to develop liver steatosis [10–12]. In addition, it has been shown that the microsomal triglyceride transporter protein (MTTP) is involved in liver steatosis. MTTP is a lipid transport protein that mediates CD1d antigen presentation, iNKT cell immunity, and lipid metabolism [13]. Because the MTTP gene likely contributes to the development of hepatic steatosis, evaluating changes in MTTP gene expression may offer clues for the early monitoring of hepatic steatosis and HCC risk among HCV patients.

The MTTP -493G/T polymorphism is characterized by allelic differences in the promoter regions of the MTTP gene, with the wild-type variant guanine (G) allele, transitioning to a thymine (T) and impacting transcription. Several studies found a significant link between changes in allelic G expression and non-alcoholic steatohepatitis (NASH) [14, 15]. However, the correlation between the T allele and liver steatosis in HCV patients is more contentious. Specifically, Rosa Zampino et al. [16] found no significant correlation between MTTP gene polymorphisms and hepatic steatosis patients presenting with hepatitis C. In contrast, Mirandola et al. [17] reported the MTTP genotype (-493G/T) T allele significantly increases the probability of liver steatosis in a population not carrying the HCV genotype 3. Later, Zampino et al. [18] also suggested a correlation between the expression of the T allele and the risk of hepatic steatosis, but solely in patients infected with HCV genotype 3. In light of these conflicting observations, it is crucial to further evaluate the relationships between MTTP genotype polymorphisms and liver steatosis associated with hepatitis C to help identifying targets for early detection and monitoring of hepatic steatosis.

The relationship between MTTP gene polymorphisms and hepatic steatosis in HCV patients remains controversial. Here, this study was the first meta-analysis evaluating the correlation between MTTP gene polymorphisms and hepatic steatosis in HCV patients. It provided a theoretical basis for subsequent studies on MTTP in the emergence of liver steatosis associated with hepatitis C.

## Materials and methods

The protocol of the present meta-analysis in the International Prospective Register of Systematic Reviews (PROSPERO) was documented (registration number: CRD42023428142).

### Search strategies

The relevant literatures were searched in some database including “Pubmed, Web of science, Embase, Cochrane library, CNKI and CBM” by using the search terms "Microsomal triglyceride transfer protein" and "Chronic Hepatitis C". The search was employed for all studies present in the databases until Augst 31, 2023. The search strategy was performed as follows: ((((((("microsomal triglyceride transfer protein") OR ("MTTP triglyceride carrier")) OR ("microsomal TG transfer protein")) OR ("MTTP protein")) OR ("Microsomal triacylglycerol transfer protein")) OR (MTTP))) AND (((((("Chronic Hepatitis C") OR ("Chronic viral hepatitis C")) OR (HCV)) OR ("Hepatitis C, Chronic ")) OR ("Hepatitis C"))). Additionally, a manual search was conducted for relevant literature citations, and discrepancies arising during the searching process were resolved.

### Criteria for inclusion of literature

#### Inclusion criteria

(1) The study type was cohort study or case–control study; (2) The study population was composed by adult (> 18 years old) patients with HCV infection; (3) The study discussed the relationship between the MTTP genotype (-493G/T) polymorphism and liver steatosis in patients with hepatitis C; (4) A liver tissue biopsy was performed to determine the presence of hepatic steatosis. The grade of steatosis followed the proportion of hepatocytes containing lipid droplets or the NAFLD activity score (NAS); (5) ORs and 95% CIs were available for the raw data of each study, or sufficient raw data to obtain ORs and 95% CIs.

#### Exclusionary criteria

(1) Study types consisted of literature reviews, conference abstracts, case reports, Cross-sectional studies or animal experiments; (2) Combined HBV or HIV infection in the study population, or other etiologies of liver disease, such as autoimmune hepatitis, alcoholic cirrhosis, and primary biliary cirrhosis (PBC); (3) Insufficient study data or no comparison between the MTTP genotype and liver steatosis.

#### Quality assessment and data extraction

The literature initially obtained from the search was screened independently by two researchers, and duplicates were removed using the EndNote X9 software. Subsequently, we read the title and abstract sections to determine if they met the inclusion and exclusion criteria. Finally, the quality evaluation of literature was performed based on “the Newcastle–Ottawa Scale” (NOS), which includes: “representativeness of the exposed cohort, ascertainment of exposure, selection of the nonexposed cohort, demonstration that outcome of interest was not present at the start of the study, comparability of cohorts on the basis of the design or analysis, assessment of outcome, long-term follow-up to allow for outcomes to occur, and adequacy of cohort follow-up”. A study was scored with one star for each item if they met the criteria. A literature score ≥ 6 stars was considered as high-quality. Subsequently, we extracted the basic data of high-quality literature as follows: Authors, Year, MTTP genotype detection method, Number of HCV steatosis and non-steatosis cases, and Gender composition. Disputes during the data extraction and quality evaluation process were resolved by another independent researcher.

#### Statistical analysis

The Stata 14.0 software was used to analyze the correlation between four MTTP genotypes, including dominant, recessive, homozygous and heterozygous gene models. The susceptibility to hepatic steatosis was estimated by calculating OR and 95% CIs. Heterogeneity was expressed using Cochran's Q and I^2^. A high heterogeneity was indicated by either *P* < 0.05 or I^2^ ≥ 50%. We used random-effects or fixed-effects models appropriately. Subgroup analysis was conducted to identify the sources of heterogeneity. Publication biases were detected using Funnel plots, the Egger's test, and the Begg's test. In addition, sensitivity analysis was also conducted to evaluate the consistency of the results. Statistical significance was determined if *P* < 0.05.

## Results

### Literature searching and screening

**Fig. 1:**
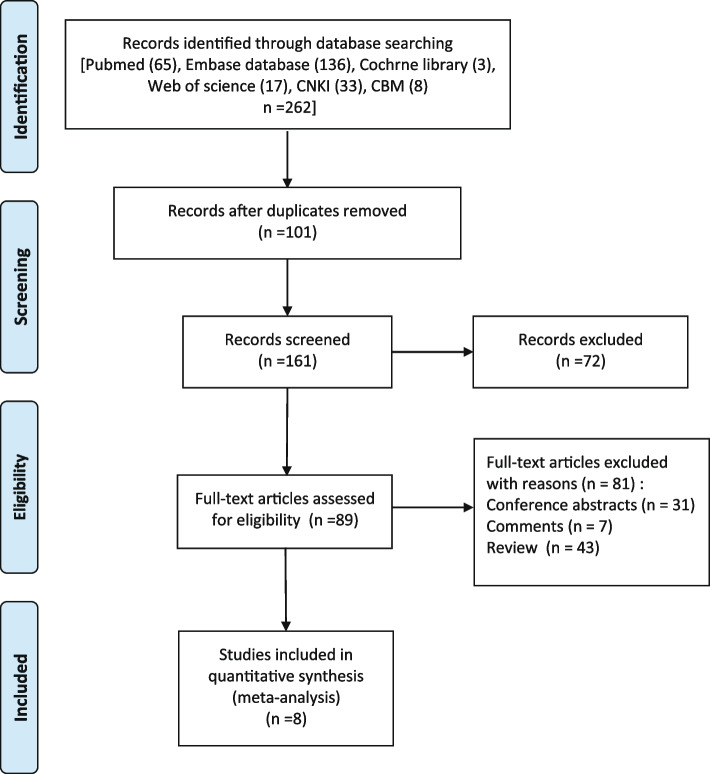
literature selection process

The strategy allowed us to retrieve 262 publications in total, including 65 from Pubmed, 136 from Embase database, 3 from Cochrne library, 17 from Web of science, 33 from CNKI, and 8 from CBM. After eliminating 101 duplicates, we screened the abstract section of the remaining 161 papers. Studies involving animal experiments or lacking information on the relationships between the MTTP genotype and the development of hepatic steatosis in HCV patients were excluded from the analyses, resulting in a final count of 89 articles. The full text of these 89 papers was meticulously analyzed and a total of 43 reviews, 31 conference abstracts, and 7 editorials were excluded. After employing these filters, we identified 8 articles meeting our selection criteria. The aforementioned literature screening strategy is shown in Fig. 1.

### Basic characteristics and quality assessment of the included studies

The 8 selected clinical studies were conducted between 2006 and 2022. In total, these studies evaluated 1535 patients with HCV, including 857 presenting with hepatic steatosis. The MTTP genotype was identified by Polymerase Chain Reaction—restriction fragment length polymorphism (PCR–RFLP) in 5 studies, and using other methods in the remaining 3 articles. Regarding the quality assessment of these clinical studies, we found one study with a score of 6, and 7 studies with a score of 7–8. This indicates a moderate-to-high quality of the included literature. These details are shown in Tables 1, and S1, respectively.

**Table 1:** Basic characteristics and NOS literature scores

Authors	year	MTTP gene	Genotyping Method	Sample size (Steatosis/Non-Steatosis)	Gender(M/F)		NOS
					Steatosis	Non-Steatosis	
Thamiris Vaz Gago Prata [19]	2022	-493G/T (rs1800591)	PCR–RFLP	125/111	47/78	56/55	8
Mariana Cavalheiro Magri [20]	2020	rs1800591	PCR–RFLP	143/147	NA	NA	6
Ersin Akgöllü [21]	2019	rs1800591	PCR–RFLP	105/39	35/70	16/23	8
Rosa Zampino [16]	2018	-493G/T	TaqMan PCR	72/32	NA	NA	7
Mariana Cavalheiro Magri [22]	2017	rs1800591	PCR–RFLP	114/125	41/73	59/66	8
E.R.F. Siqueira [23]	2012	-493G/T	PCR DNA sequencing	93/45	NA	NA	8
Silvia Mirandola [17]	2009	-493G/T	PCR–RFLP	166/132	92/74	75/57	8
Jean Michel Petit [24]	2006	-493G/T	PCR DNA sequencing	39/47	NA	NA	8

*PCR–RFLP* Polymerase Chain Reaction—restriction fragment length polymorphism

### MTTP dominant model (GT + TT vs GG) and susceptibility to hepatic steatosis associated with hepatitis C

**Fig. 2:**
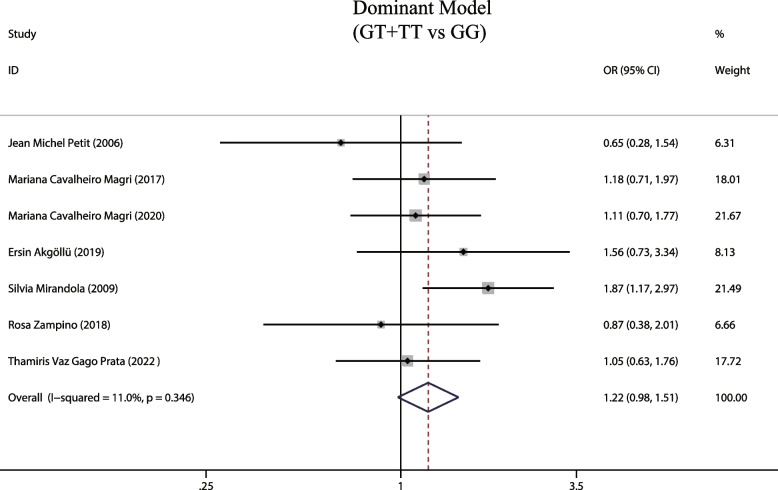
The dominant model and susceptibility to HCV hepatic steatosis

**Fig. 3:**
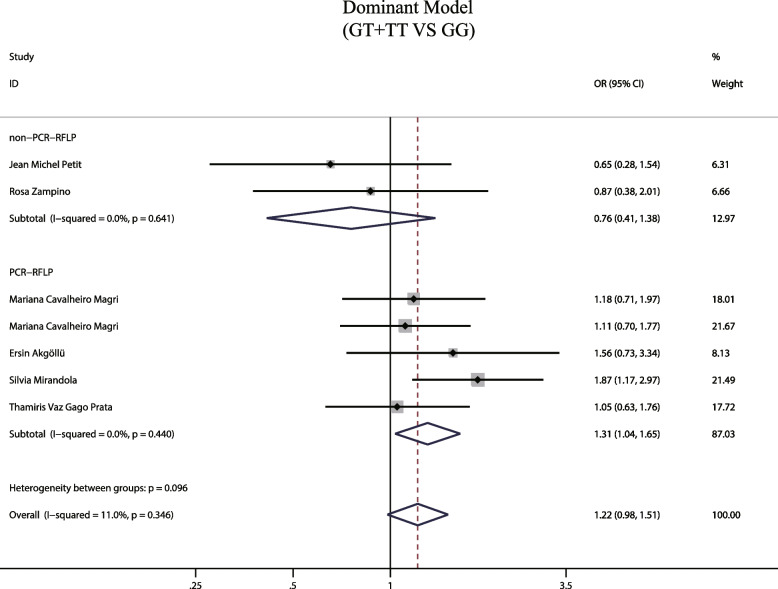
The dominant model and susceptibility to HCV hepatic steatosis (subgroup analysis according to MTTP gene assay)

A total of 7 articles reported a positive relationship between the MTTP genotype -493G/T dominant model (GT + TT) and hepatic steatosis during HCV infection. Due to the low heterogeneity observed (I^2^ = 11%, *P* = 0.346), we employed a fixed-effects model that showed the dominant gene mutation model did not increase risk of hepatic steatosis (OR = 1.22, 95% CIs: 0.98–1.51, *P* = 0.076, Fig. 2). A subgroup analysis was performed considering the methodological differences used to detect the MTTP gene across the various studies. This analysis indicated the dominant mutation model increases the risk of liver steatosis in HCV patients in the 5 studies using PCR–RFLP as the detection method (OR = 1.306, 95% CIs: 1.035–1.646, *P* = 0.024, Fig. 3), but found no significance in studies employing other detection methods (*P* = 0.362). Since, only two studies were included, further assessment is necessary to confirm these observations. Our findings thus suggest the detection method may introduce biases in the results, indicating the need for further analysis of the impact of gene detection methods on the study of the correlation between MTTP and hepatic steatosis associated with hepatitis C.

**Fig. 4:**
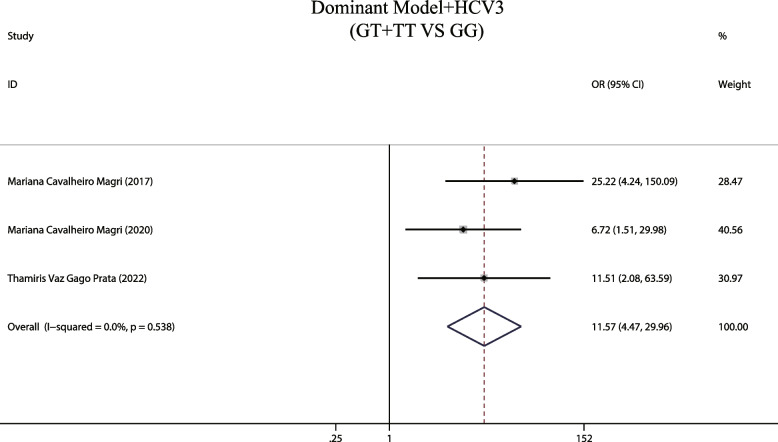
The dominant model and susceptibility to HCV genotype 3 hepatic steatosis

In addition, the result also found that HCV genotype 3 combined with the MTTP gene dominant model mutation results in an 11.57-fold increase in the risk of development of liver steatosis (OR = 11.57, 95% CIs: 4.467 -29.962, *P* < 0.001, Fig. 4). These observations are consistent with previous findings by Silvia [17].

### MTTP gene recessive model (TT vs GG + GT) and susceptibility to hepatic steatosis associated with hepatitis C

**Fig. 5:**
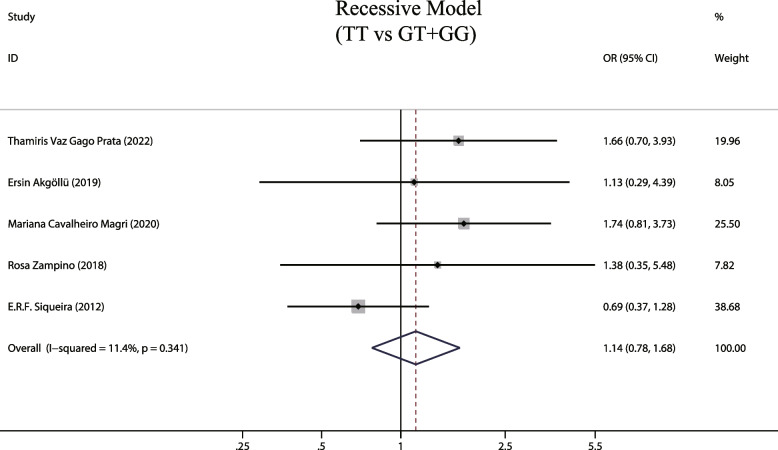
The recessive model and susceptibility to HCV hepatic steatosis

**Fig. 6:**
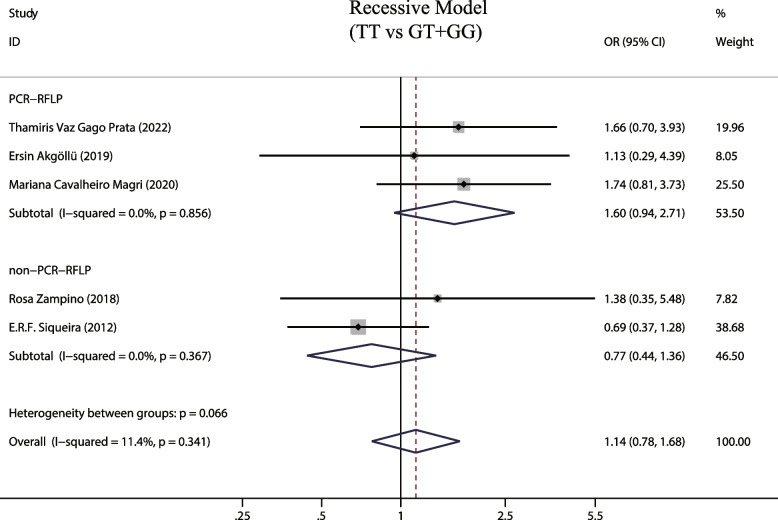
The recessive model and susceptibility to HCV hepatic steatosis (subgroup analysis according to MTTP gene assay)

**Fig.7:**
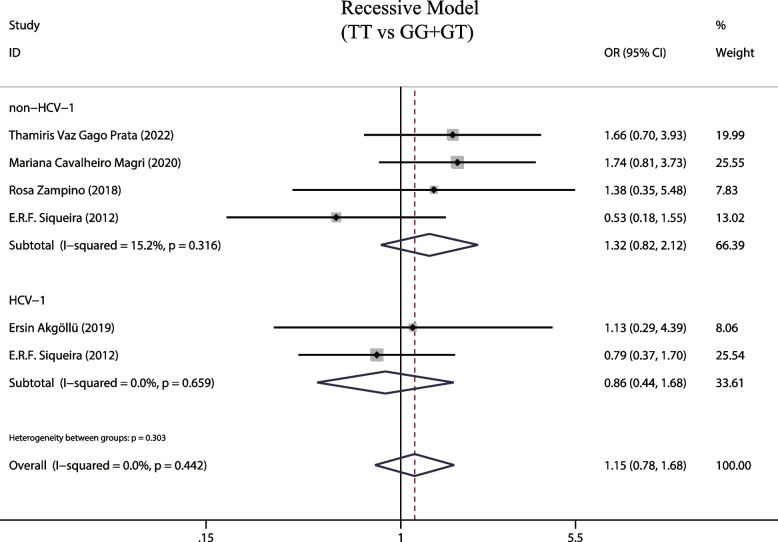
The recessive model and susceptibility to HCV hepatic steatosis (subgroup analysis according to HCV genotype)

A total of 5 studies examined the effects of MTTP gene recessive models and susceptibility to hepatic steatosis following hepatitis C infection. A fixed-effects model was employed due to the observed low heterogeneity between studies (I^2^ = 11.4%, *P* = 0.341). The MTTP genotype-493G/T recessive model did not show a correlation with susceptibility to hepatic steatosis (OR = 1.142, 95% CIs: 0.78–1.68, *P* = 0.5, Fig. 5). In addition, subgroup analysis was on different detection methods of MTTP genotype and no increased risk of liver steatosis was found (*P* > 0.05, Fig. 6). Finally, we also analyzed the relationship between MTTP genotypes and susceptibility to hepatic steatosis in different HCV genotypes, but found no increased risk in patients infected by the HCV genotype 1 and harboring MTTP recessive model mutations (OR = 0.862, 95% CIs: 0.443–1.676,* P* = 0.661, Fig. 7).

### Homozygous and heterozygous MTTP gene models and susceptibility to hepatic steatosis associated with hepatitis C

**Fig. 8:**
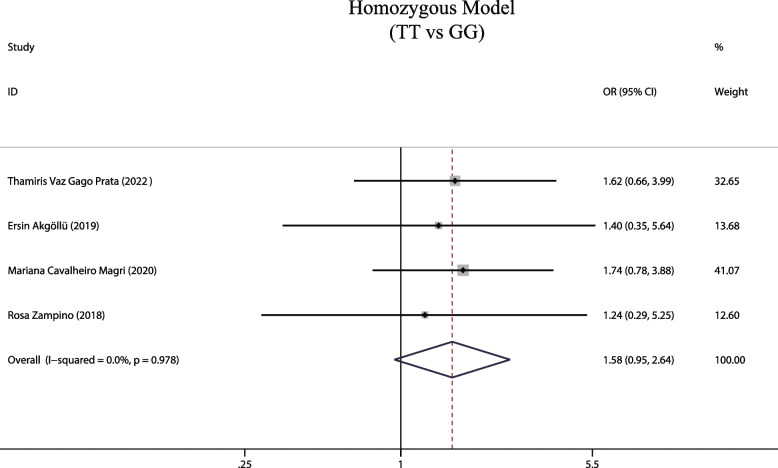
The homozygous model and susceptibility to HCV hepatic steatosis

**Fig. 9:**
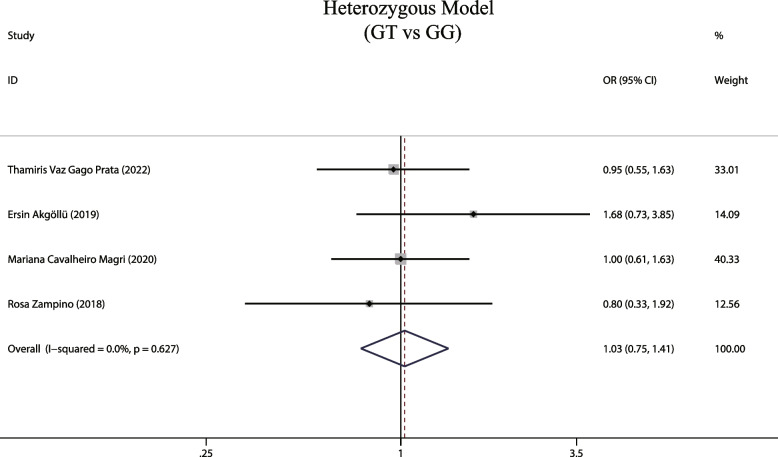
The heterozygous model and susceptibility to HCV hepatic steatosis

A total of 4 studies investigated the relationship between homozygous and heterozygous models and susceptibility to hepatic steatosis associated with hepatitis C. Our fixed-effects model analysis showed both homozygous and heterozygous models are not associated with the risk of development of hepatic steatosis [(OR = 1.581, 95% CIs: 0.946–2.644, *P* = 0.081, Fig. 8); (OR = 1.029, 95% CIs: 0.753–1.405, *P* = 0.86, Fig. 9)].

### Publication biases and sensitivity analysis

**Fig. 10:**
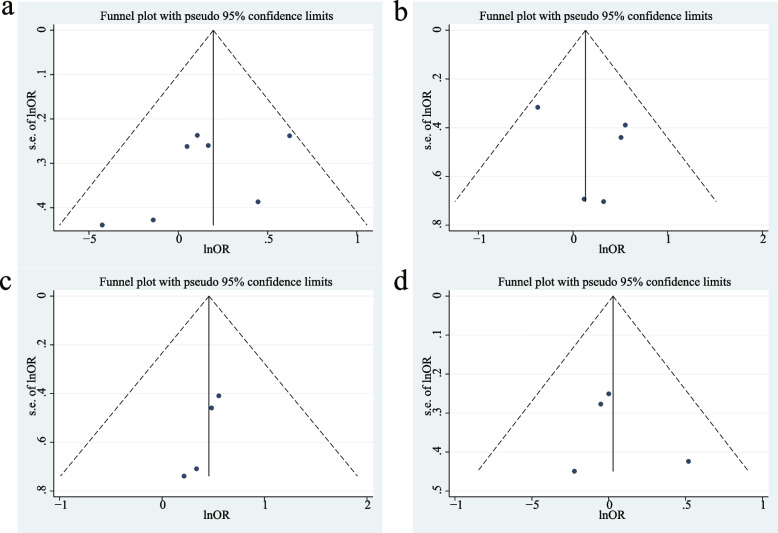
Funnel plots of different MTTP genotype models: **a** A dominant model; **b** A recessive model; **c** A homozygous model; **d** A heterozygous model

**Fig. 11:**
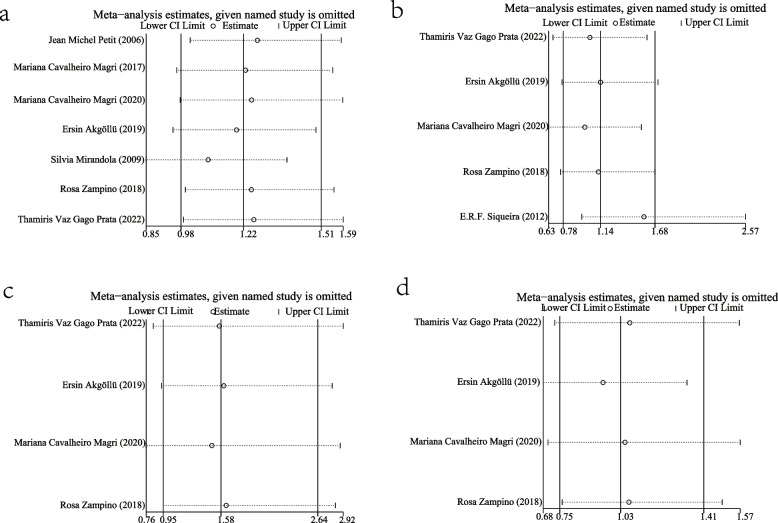
Sensitivity analysis of different MTTP genotype models: **a** A dominant model; **b** A recessive model; **c** A homozygous model; **d** A heterozygous model

After analyzing the data for each MTTP gene model, the Funnel plots, the Egger's tests and the Begg's tests were employed to identify potential biases in publications. The results showed that a largely symmetrical distribution of Funnel plots, except for the dominant model (Fig. 10). Subsequent quantitative analysis on the dominant model using the Egger’s test and the Begg’s test found no significant publication biases (*P* > 0.05, Table 2). In the homozygous model, the Egger’s test indicated possible bias, but this was not confirmed by the Begg’s test or the Funnel plots. Then the sensitivity analysis was performed by excluding articles one-by-one. The OR and 95% CIs obtained from this analysis did not differ significantly from the final results for each gene model (Fig. 11). Overall, the results suggest this meta-analysis is not affected by publication biases.

**Table 2:** Egger’s and Begg’s tests performed for different genetic models

Genetic model	*P*_*E*_	*P*_*B*_
Dominant model	0.263	0.133
Recessive model	0.488	1
Homozygous	0.029	0.089
Heterozygous	0.692	0.734

*PE* *P* value of the Egger’s linear regression test, *PB* *P* value of the Begg’s rank correlation test

## Discussion

Patients presenting with hepatitis C infection have a higher susceptibility to liver steatosis, which is closely linked to late-stage cirrhosis and liver cancer. This meta-analysis discussed the relationship between MTTP genotype polymorphisms and susceptibility to liver steatosis in HCV patients. This study began with a comprehensive screening based on strict inclusion and exclusion criteria, and subsequently included novel publications from recent years. The result found the dominant model (GT + TT) is positively correlated with an increased risk of liver steatosis associated with hepatitis C. Notably, the risk of liver steatosis increased significantly when the HCV genotype 3 was combined with a dominant model of MTTP mutations, which is consistent with the findings by Tao Cai [25, 26]. In contrast, no correlation to hepatic steatosis susceptibility was observed in other mutation models. These observations indicate the expression of the T allele in the MTTP genotype (-493G/T) may lead to increased susceptibility to hepatic steatosis in patients carrying the HCV genotype 3.

The MTTP genotype (-493G/T) polymorphism alters the promoter region and transcriptional activity of the MTTP gene [27]. Moreover, multiple studies showed the T allele can increase transcriptional activity of the MTTP gene, while the G allele has the opposite effect. André P et al. [28] found the G allele is more common in NASH, and that the GG homozygous genotype combined with more severe liver steatosis is associated with the suppression of MTTP expression, leading to hepatic lipid accumulation. This study confirmed the T allele is related to the development of liver steatosis in HCV patients. However, it should be noted that the effect of the T allele on hepatitis C liver steatosis does not directly involve MTTP-mediated impacts on lipid metabolism. Instead, enhanced MTTP transcriptional activity leads to the assembly and secretion of viral particles. R. Zampino [18] showed that patients carrying the HCV genotype 3 combined with the MTTP T allele express higher levels of viral RNA. This suggests the expression of the T allele and MTTP transcription are associated with HCV viral particle production and replication. HCV viral particles contain apolipoproteins, whereby their production and secretion depend on very low-density lipoprotein (VLDL) and other types of apolipoproteins [29, 30]. MTTP is involved in the assembly and secretion of VLDL and ApoB [31]. Consequently, enhanced transcriptional activity of MTTP produces higher levels of VLDL, increasing the ability to assemble, produce and replicate HCV virus particles [32, 33]. High concentrations of the HCV core protein in later stage hepatitis C chronic patients promote liver lipid accumulation by inhibiting the expression of the MTTP protein expression. In the HCV genotype 3, the core protein has the strongest effect on lipid accumulation by inhibiting the expression of the MTTP protein and preventing the secretion of lipoproteins [7, 34]. Accordingly, patients with the HCV genotype 3 have an increased risk of liver steatosis when carrying the dominant MTTP genotype [26]. Since the expression levels of the MTTP gene dynamically changes during the early and late stages of HCV infection, assessing the risk of liver steatosis depends on monitoring changes in MTTP genotype expression during early HCV infection.

### Strengths and limitations

This meta-analysis has several advantages. First, this study is the first to explore the relationship between the MTTP genotype and hepatic steatosis susceptibility in HCV patients. Second, this study includes a systematic and comprehensive literature search, which was included the latest findings on the MTTP gene and HCV hepatic steatosis susceptibility. Third, there was low heterogeneity in each of the groups included in this study, and no impacts arising from publication biases. Fourth, the effect of the MTTP gene on hepatic steatosis of different HCV genotypes was also discussed through subgroup analysis. Finally, this study showed that the MTTP genotype polymorphism is related to hepatic steatosis during HCV infection, which may become an early indicator of hepatic steatosis.

This study is limited by the inclusion of a small number of literature sources, as it only analyzed the relationship between the MTTP genotype and hepatic steatosis susceptibility in patients with HCV genotypes 1 and 3. The correlation between MTTP gene polymorphisms combined with other HCV genotypes, and the resulting susceptibility to hepatic steatosis were not explicitly investigated. Further research is thus necessary to explore the potential impact of various HCV genotypes and MTTP gene mutations on hepatic steatosis susceptibility. Additionally, subgroup analyses found MTTP dominant model mutations may increase the risk of liver steatosis in HCV patients. However, due to differences in the detection methods, these findings cannot conclusively exclude false-positive results. More rigorous studies are needed to verify the mechanistic link between MTTP genotype polymorphisms and liver steatosis in HCV patients.

## Conclusions

In conclusion, this meta-analysis further confirmed that dominant mutations in the T allele of the MTTP genotype (-493G/T) increase susceptibility to hepatic steatosis in patients presenting with the HCV genotype 3.

### Supplementary Information

**Additional file 1:** **Table S1**. NOS for included studies

## Data Availability

The datasets generated during the current study are available from the corresponding author on reasonable request.
