# Supplementary material for: Association between MTTP genotype (-493G/T) polymorphism and hepatic steatosis in hepatitis C: a systematic review and meta-analysis
Source: Lipids Health Dis. 2023 Sep 19;22:154. doi: 10.1186/s12944-023-01916-x (PMC10507831; doi:10.1186/s12944-023-01916-x)
Supplement: Supplementary file 1 — Additional file 1: Table S1. NOS for included studies [file 12944_2023_1916_MOESM1_ESM.pdf]

Better Papers Faster™

Language Editing Certification

To whom it may concern:

The manuscript, entitled “MTTP genotype (-493G/T) polymorphism and susceptibility to hepatic steatosis in hepatitis C： a meta-analysis”, has been professionally edited for English usage, grammar, spelling, and punctuation by a native English speaker and a skilled professional editor. Therefore, we would appreciate if any comments regarding language are as specific as possible.

Genesis Technology Communication (Beijing), Co., Ltd.

2023.5.22

Better Papers Faster
